# Nanostructured lipid carriers: versatile oral delivery vehicle

**DOI:** 10.4155/fsoa-2016-0030

**Published:** 2016-07-15

**Authors:** Neelam Poonia, Rajeev Kharb, Viney Lather, Deepti Pandita

**Affiliations:** 1 Department of Pharmaceutics, JCDM College of Pharmacy, Sirsa 125055, Haryana, India; 2 Department of Pharmaceutical Chemistry, CT Institute of Pharmaceutical Sciences, Jalandhar 144020, Punjab, India; 3 Department of Pharmaceutical Chemistry, JCDM College of Pharmacy, Sirsa 125055, Haryana, India

**Keywords:** controlled drug release, gene/drug delivery, nanoparticles

## Abstract

Oral delivery is the most accepted and economical route for drug administration and leads to substantial reduction in dosing frequency. However, this route still remains a challenge for the pharmaceutical industry due to poorly soluble and permeable drugs leading to poor oral bioavailability. Incorporating bioactives into nanostructured lipid carriers (NLCs) has helped in boosting their therapeutic functionality and prolonged release from these carrier systems thus providing improved pharmacokinetic parameters. The present review provides an overview of noteworthy studies reporting impending benefits of NLCs in oral delivery and highlights recent advancements for developing engineered NLCs either by conjugating polymers over their surface or modifying their charge to overcome the mucosal barrier of GI tract for active transport across intestinal membrane.

## Abstract (lay abstract)

**Lay abstract:** Oral administration of drugs is considered to be a convenient route; however, various drugs that are insoluble in water or unable to permeate across GI tract membrane cannot be delivered by this route. To deliver them effectively, various lipid carriers have been widely explored by researchers. Lipid carriers encapsulate drug inside them and deliver them effectively via the oral route. Also, encapsulation of drug protects them from degradation inside GI tract and safely delivers them to the site of action. This review summarizes application of lipid carriers, in other words, nanostructured lipid carriers, in eradicating these problems, with suitable examples.

**Figure 1.**
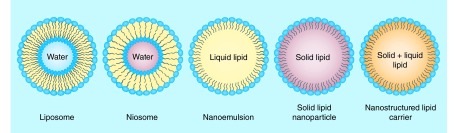
**Various lipid-based systems explored for drug delivery applications.**

**Figure 2.**
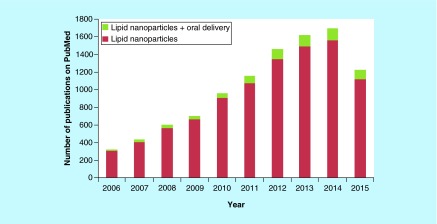
**Total number of publications on PubMed respective to the term ‘lipid nanoparticles’ and ‘lipid nanoparticles + oral’.**

**Figure 3.**
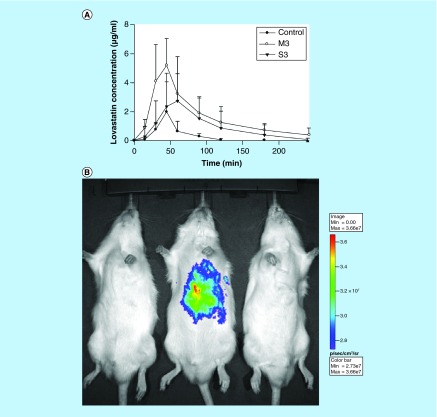
**Effect of type of emulsifier on oral bioavailability of lovastatin.** **(A)** Mean plasma concentration of lovastatin–time curves after oral administration of lovastatin to rats at a dose of 100 mg/kg from a control solution and nanostructured lipid carriers (NLC)s with M3, and S3. **(B)** Bioluminescence imaging of representative animals at 10 min following oral administration of sulforhodamine B in a control solution, NLCs with M3 and NLCs with S3 (left to right). M3: Myversol; S3: Soyabean phosphotidyl choline. Reproduced with permission from [19] © Elsevier (2010).

**Figure 4.**
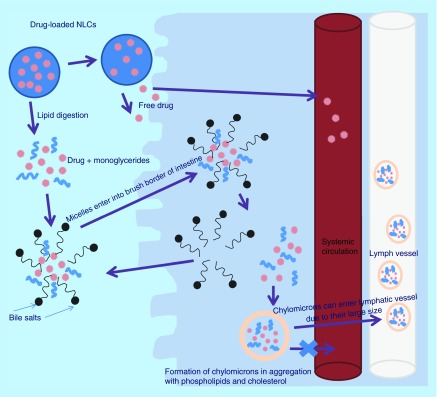
**Mechanism of nanostructured lipid carriers disposition.** NLC: Nanostructured lipid carrier.

**Figure 5.**
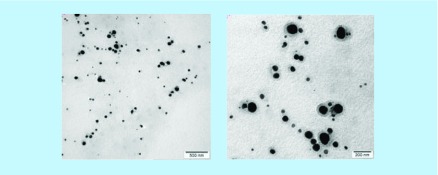
**Transmission electron miroscopy of nanostructured lipid carriers.** Reproduced with permission from [37] © Elsevier (2016).

**Figure 6.**
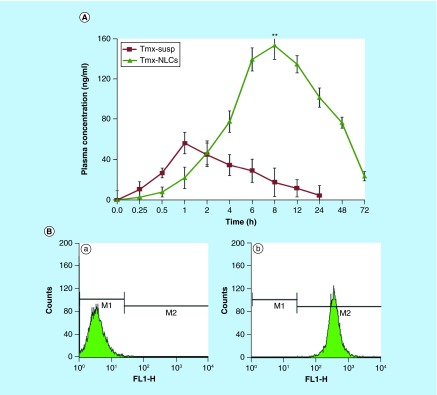
**Pharmacokinetics and cellular uptake of tamoxifen-loaded nanostructured lipid carriers.** **(A)** Plasma concentration–time profiles of tamoxifen suspension and tamoxifen-loaded NLCs after single oral administration of 10 mg/kg to rats. **(B)** Flow-cytometric analysis for cell uptake studies. **(a)** Histogram of untreated control cells, and **(b)** histogram of cells after 3 h incubation with coumarin-6-labeled tamoxifen-loaded NLCs. FL1-H: Fluorescence intensity histogram; NLC: Nanostructured lipid carrier; susp: Suspension; Tmx: Tamoxifen. Reproduced with permission from [46] © Elsevier (2013).

**Figure 7.**
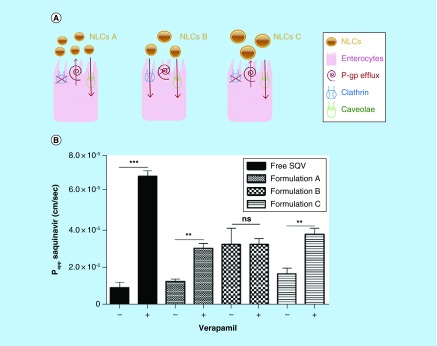
**Transport of saquinavir-loaded nanostructured lipid carriers across the intestinal barrier.** **(A)** The transport mechanisms used by the different nanostructured lipid carriers formulations. **(B)** Permeability (P_app_) values for free saquinavir and the saquinavir-loaded nanostructured lipid carriers after 2 h of incubation with 100-μM verapamil, a P-gp inhibitor. Formulations with no inhibition were considered as controls (n = 3). ** p < 0.01; *** p < 0.001. –: Absence of verapamil; +: Under verapamil inhibition. NLC: Nanostructured lipid carrier; ns: No significance; SQV: Saquinavir. Reproduced with permission from [34] © Elsevier (2013).

**Figure 8.**
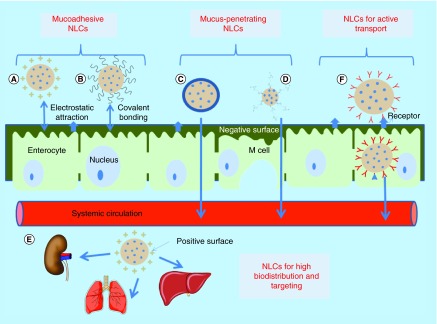
**Various engineered nanostructured lipid carriers.** **(A)** Positive-charged NLCs having electrostatic attraction with negatively charged intestinal cells, **(B)** thiomer-conjugated NLCs covalently binds with cysteine group of mucus, **(C)** neutral charge NLCs breaks through mucus layer, **(D)** PEG-coated NLCs breaks through mucus layer, **(E)** positive charged NLCs for high biodistribution and targeting and **(F)** ligand-conjugated NLCs bind with receptors on intestinal cells and undergo endocytosis. NLC: Nanostructured lipid carrier.

**Figure 9.**
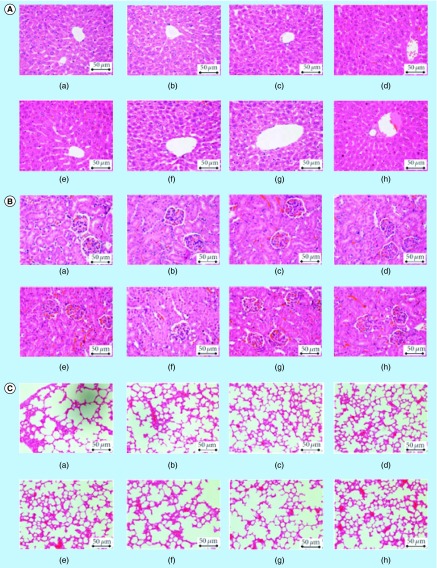
**Evaluation of potential toxicity of zerumbone-loaded nanostructured lipid carriers in BALB/c mice model through histopathology of tissues.** **(A)** Liver, **(B)** kidney and **(C)** lung histograms of female BALB/c mice treated orally, with **(a)** water (control), **(b)** olive oil (vehicle), **(c & d)** zerumbone at concentrations 100 and 200 mg/kg, **(e & f)** blank nanostructured lipid carriers at concentrations 100 and 200 mg/kg and **(g & h)** zerumbone-loaded nanostructured lipid carriers at concentrations 100 and 200 mg/kg for 14 days. No sign of toxicity was observed in the organs of these mice (400 × magnification). Reproduced with permission from [40] © Creative Commons Attribution License.

The oral drug administration route provides a valuable option for treating various deadly diseases because of its several advantages like patient compliance, cost–effectiveness and ease of administration – and is regarded as the most commonly accepted route for drug administration. It is also highly preferred for chronically administered agents, such as anti-tumor, antidiabetic and antihypertensive agents. Drug candidates, which are stable in gastric environment, possess adequate hydrophilic lipophilic balance to cross the intestinal epithelium membrane with the absence of the significant GI tract (GIT), irritation and toxicity signs are ideal candidates for oral delivery [1]. Unfortunately, more than 40% of drugs coming out of the drug discovery and development processes are not suitable for oral delivery due to their hydrophobic nature and present poor oral bioavailability, that is, insufficient drug is presented to the site of action with subsequent lack of pharmacological action. Several other barriers are also encountered with the oral route, which are responsible for comparatively poor plasma levels of orally administered drugs *viz.* pitiable permeability across the gastrointestinal membrane, first pass metabolism, drug expulsion via intestinal drug transporter, that is, P-glycoprotein (P-gp) and variability due to food effects [1,2]. Therefore, there is a prescient necessity for researchers to make advancements in oral drug delivery systems and the above mentioned factors need considerations for providing the desired therapeutic outcomes.

For bioavailability enhancement, the researchers have attempted various approaches to overcome the challenges associated with oral delivery, such as nanosizing of the drug molecules, salt formation, prodrug synthesis and encapsulation of drugs in nanosized carriers, such as polymeric micelles, nanoparticles, liposomes, emulsions, etc. [1,3–4]. Various chelating agents and ionic polymers have also been explored by researchers for enhancing absorption of drug molecules [5]. Also, the studies have shown that P-gp blockers can also be employed for enhancement of oral bioavailability [6,7]. In the past few decades, lipid-based drug delivery systems have offered a glance of hope for their favorable effects on absorption of encapsulated drugs. Figure 1 represents various lipid-based systems, which are being explored for drug delivery applications. PubMed shows huge published literature (9461) from 2006 to date when searched for the term ‘lipid nanoparticles’ and approximately 7.3% of those were found under ‘lipid nanoparticles in oral delivery’, thus depicting huge interest of researchers for employing them as a promising vehicle for oral delivery (Figure 2). Although numerous exhilarating results have been obtained with conventional lipid-based systems, in other words, micelles, liposomes and nanoemulsions, but reports suggest that these systems are susceptible to degradation during storage and in the GIT due to the acidic environment of the stomach, intestinal enzymes and bile salts [8–10]. To overcome such limitations, Muller *et al*. extensively worked on biocompatible and biodegradable solid lipids to develop solid lipid nanoparticles (SLNs) in early 1990 and thus resolved the stability and toxicity issues associated with the conventional lipid-based delivery systems [4]. However, SLNs also suffered from various shortcomings, such as poor drug loading capacity and drug expulsion during storage [9,11]. The drug leakage problem in them appeared due to their transformation from high-energy modification into more ordered modification (β) on storage [12]. Modified lipid nanoparticles, also termed as nanostructured lipid carriers (NLCs), came into picture in early 2000 to modulate the physical state and drug loading capacity of SLNs [13].

NLCs possess unique characteristics and are formulated using a combination of solid and liquid lipids where less ordered structures are produced, which offer the firmer inclusion of the drug molecules within the matrix during the shelf life [14,15]. It has been consistently reported that the increased entrapment efficiency in NLCs is attributed to the structural parity of two lipids in NLCs that results in imperfections in their structure while solidification provides higher space for accommodation of drugs [11]. Also, the higher entrapment efficiency is due to higher solubility of drugs in liquid lipids in comparison to solid lipids [16]. Higher pay load capacity along with long shelf storage stability thus makes NLCs as an advanced carrier in comparison to other conventional lipid-based systems. Further, NLCs have the feasibility of incorporating both hydrophilic and lipophilic drugs. In addition to this, they may provide sustained release of drugs and target them to the site of action. Various studies have proven that this nanoplatform has shown improvement in oral bioavailability of drugs via promoting their intestinal absorption [17]. This system has also shed a light of hope for treatment of chronic diseases due to its modulation with drug efficacy and sustained for longer periods. This review primarily focuses on the composition and fabrication process of NLCs influencing their properties and their role as a delivery vehicle in the enhancement of oral bioavailability of various drugs and advancements made to further improve its performance.

## NLCs versus SLNs

As mentioned previously NLCs overcome the disadvantages associated with SLNs, in other words, they provide higher drug loading, faster release rate and storage stability due to a use of blend of solid lipid and liquid lipid in their formulations. Table 1 gives an account of the various properties of SLNs and NLCs. Several comparative studies have been documented in literature in which NLCs have served as a better carrier than SLNs. For example, in a study between SLNs and NLCs of simvastatin, it was observed that entrapment efficiency of the drug was improved in the case of the later (93.33%) when compared with SLNs (75.81%) due to creation of extra space for loading by liquid lipid [18]. Also, the *in vitro* release patterns in both SLNs and NLCs were similar but NLCs displayed high-percent cumulative drug release in comparison to SLNs in 55 h. A lesser mobility of drug in SLNs (a crystallized system) in comparison to NLCs (disordered arrangement) was responsible for the slower release of drug. Differential scanning calorimetric analysis showed decreased recrystallization index of NLCs in comparison to solid lipids and physical mixture of solid lipid and liquid lipid favoring the formation of disordered arrangement and reduced capacity of solid lipids to recrystallize suggesting their higher long-term stability. The results of *in vivo* studies also suggested NLCs to be superior as they exhibited 2.29-fold increase in oral bioavailability when administered to mice. Similar results were also noted with lovastatin-loaded NLCs. Study of partitioning behavior of lovastatin in pure solid lipid and mixtures of solid lipid and liquid lipid also depicted higher partitioning of drug in the lipid phase consisting of a mixture of solid lipid (Precirol^®^ ATO 5) and liquid lipid (squalene) thus suggesting that higher solubility of drug was favored by the presence of liquid lipid [19]. Another report on progesterone [20] and domperidone-loaded [21] SLNs and NLCs also suggested the NLCs as a better vehicle in respect of drug loading and release rate.

## Fabrication of NLCs

NLCs are generally composed of solid lipid, liquid lipid, emulsifiers and water. They are fabricated using various methods, such as emulsification-sonication, solvent injection, high-pressure homogenization, microemulsion and solvent diffusion techniques that have been reviewed elsewhere [11,13,22]. Tables 2–6 provide an overview of the drugs encapsulated in NLCs for oral delivery on the basis of the method of preparation employed. These systems possess commercial potential, as their method of production specifically the high-pressure homogenization and microemulsion technique provides ease of scalability [13]. NLCs dispersions obtained by the aforementioned methods may further be converted to powdered form via freeze-drying process for physical and chemical stabilization of nanoparticles. Freeze drying process finds wide applicability due to higher yield and low moisture content in the final product [23]. Spray drying may also be used as an alternative technique that also is cost effective in comparison to the former technique [24].

Literature reveals that composition and processing parameters employed for fabricating NLCs play a vital role in defining their particle size, percent entrapment efficiency and drug release profile and have been manipulated by various formulation scientists to obtain desired characteristics. Tailoring these properties can be advantageous for obtaining high therapeutic levels of drug in blood plasma. For instance, Muchow *et al*. observed that small sized NLCs (200 nm) showed higher AUC values in comparison to higher sized NLCs (600 nm) when administered orally to rats, this was attributed to their higher mucoadhesion capability in the body [36].

### Lipids

Solid lipid and liquid lipids, the major constituents of NLCs, ideally should be biocompatible, biodegradable, chemically stable and not possess any toxic effects. The selection of lipids for formulating NLCs is usually done on the basis of solubility of the drug in the lipids as it has a direct impact on drug entrapment and loading efficiency of NLCs [17]. For instance, Mendens *et al*. revealed that higher solubility of miconazole in lipid provided its better encapsulation in NLCs. In this study, the authors screened various solid lipids (Gelucire^®^ 43/01, Precirol^®^ ATO 5, Compritol^®^ 888 ATO, Gelucire^®^ 39/01, Gelucire^®^ 50/13, Suppocire^®^ CM, inwitter^®^ 900 and stearic acid) and liquid lipids for formulating miconazole-loaded NLCs. Gelucire 43/01 was selected as solid lipid because it presented highest solubility for miconazole. Among two liquid lipids, in other words, Migilol^®^ 812 and Capryol^®^ PGMC, short listed on the basis of solubility study, capryol PGMC was employed for the fabrication of NLCs as it had higher compatibility with solid lipid selected [49]. Similarly, saturation solubility of lercanidipine HCl was determined in various solid lipids, liquid lipids and surfactants and all components were selected on the basis of highest solubility for assuring better entrapment efficiency. In addition to selecting best lipid on the basis of solubility, compatibility studies between lipid and drug are also necessary to produce stable NLCs. Phase separation has been reported for various combinations of solid and liquid lipids and the combinations showing no separation till 24 h after mixing are selected for formulating stable NLCs [55]. Hydrophilic drugs have also been successfully entrapped in lipids, for example montelukast showed high entrapment efficiency (96.13%) with lipids, in other words, Precirol ATO 5 and Caproyl 90 in which it had highest solubility [53]. Kumbhar *et al*. carried out ^1^H NMR analysis to determine any interaction between lipid and bicalutamide as it contains functional groups capable of accepting and donating protons that may interact with the lipids during the preparation of NLCs leading to incompatibility [27]. FTIR analysis has also been used to confirm no interactions between drug and excipients selected [53].

Amount of liquid lipid is another parameter that plays a significant role in tailoring particle size and release rate as it leads to reduction in viscosity and surface tension of the system that provides smaller sized NLCs, which in turn provides high surface area, promoting higher percent cumulative drug release as demonstrated by Tiwari *et al*. [56]. Chen *et al*. also reported that the particle size of NLCs was significantly affected by the presence of liquid lipid. Pure solid lipids generated higher sized particles, whereas continuous increment in liquid lipid content led to decrease in particle size due to decrease in viscosity and higher molecular mobility [57]. A similar observation was also noted by Tran *et al*. wherein the continuous addition of Labrafil M 1944CS to Compritol 888 ATO led to the formation of small-sized NLCs. Better entrapment efficiency was also obtained with the addition of liquid lipid due to enhanced solubility of drug facilitated by addition of liquid lipid [47].

Various lipids/oils obtained from plants are also gaining popularity in the pharmaceutical field for formulating lipid nanoparticles. For instance, tripterine was encapsulated in lipid derived from an edible plant, in other words, broccoli by solvent diffusion method and enhanced oral bioavailability was achieved in comparison to Precirol ATO 5-based lipid nanoparticles and drug suspension suggesting the applicability of plant-based lipids [58]. Also, siberian pine seed oil and fish oil have been employed as liquid oils in formulating stable NLCs [59,60]. In a study, vegetable oils provided the synergistic antioxidant effect of green tea extract when used as a liquid lipid for fabrication of NLCs composed of cetyl palmitate and glyceryl stearate [61]. There are, very few reports on exploitation of plant-derived lipids for fabrication of lipid nanoparticles and it still needs to be explored in encapsulating bioactives and their safe and effective delivery.

Amount of total lipid matrix is also known to affect particle size and entrapment efficiency of NLCs, in other words, an enhancement in both characteristics was observed with increasing the lipid matrix. The higher viscosity of the system is held responsible for growth in particle size, whereas reduction in drug escaping tendency due to higher lipid content results in higher entrapment efficiency. Thus, selection of optimal lipid content is crucial while formulating NLCs with desired properties [62].

### Emulsifiers

Emulsifiers are incorporated into NLCs formulations for dispersing one immiscible phase into another during their fabrication. They also provide long term stabilization against aggregation via forming a coat over the surface of NLCs [63]. The emulsifiers tend to decrease the interfacial tension between two phases (lipid solvent and water), which in turn increases the surface area of lipid droplets generating smaller particles [64]. Different release kinetics profile and entrapment efficiency values were obtained while using different types and amounts of emulsifiers. For example, in a study, NLCs of size 20 nm were obtained using 2% of Tween 20, whereas the increased concentration of same surfactant (3%) showed higher particle size (100 nm) [41]. This could be attributed to the fact that emulsifiers could reduce the interfacial tension till a specific concentration above which excess coating of particles reduced the ζ-potential leading to agglomeration of the particles [65]. In another study, it was seen that NLCs composed of myversol surfactant provided a higher encapsulation efficiency in comparison to soyabean phosphatidylcholine surfactant due to formation of a rigid interfacial film over the interface during emulsification capable of accommodating the drug with higher efficacy [19]. Also, higher release was observed due to presence of fatty acids in myversol that favored formation of mixed micelles that facilitated faster diffusion of drug during incubation period in the *in vitro* release study. Improved characteristics of myversol system resulted in higher bioavailability of lovastatin-loaded NLCs in orally administered rats (Figure 3A). *In vivo* bioluminescence imaging results were also in their concordance showing higher accumulation of NLCs composed of myversol surfactant (Figure 3B) suggesting the applicability of modified release rate and entrapment efficiency in enhancing therapeutic efficacy. The hydrophobic solid lipid, in other words, Precirol ATO 5 (HLB = 2) used for formulation of NLCs resulted in the slow release rate of bicalutamide, but the presence of hydrophilic surfactant with high HLB ≥18 modulated faster release of drug from NLCs [27]. Thus, selection of emulsifier and its concentration is an important parameter during formulation of NLCs to develop an effective delivery system having defined particle size, narrow size distribution and guaranteeing a more predictable and specific drug release. Few studies have employed a blend of surfactants to obtain sufficient viscosity for further enhancing stability of NLCs [39,66]. In addition, emulsifiers also provide other beneficial properties to NLCs, such as Solutol HS 15 having a higher affinity for P-gp can be beneficial for effective delivery of P-gp substrate drugs, such as vinpocetine, etoposide, etc. and they are also known to disturb the intestinal membranes leading to higher permeability of NLCs [39,67].

### Miscellaneous parameters

Method of preparation also plays a critical role in the characteristics of NLCs. Ranpise *et al*. observed that smaller particle size with a high entrapment efficiency was obtained using a solvent evaporation method whereas ultrasonification method yielded higher sized particles with a low-encapsulation efficiency [55]. Furthermore, improved entrapment efficiency has been obtained using the melt-emulsification technique instead of solvent-diffusion method as solubility of drug in the aqueous phase is promoted during the diffusion of organic solvent in later method, which leads to decreased encapsulation of drug and is found to be more accumulated on the surface of NLCs [20]. Lutein-loaded NLCs showed subsequent decrease in particle size with increasing sonication time from 2 to 10 min as higher ultrasonic energy facilitated dispersion of lipid in small-sized droplets [68]. The ratio of oil and water phase was also observed to affect encapsulation of drug in NLCs. In this study, an increase in water phase provided higher encapsulation attributed to the decreased aggregation of particles facilitated by higher space [62]. Thus, the results of all these investigations emphasize on the necessity of judicial selection of various parameters for formulating NLCs with optimal characteristics.

## Mechanism of NLCs disposition

Various mechanisms have been proposed for NLCs disposition inside the body either via their selective uptake through lacteals or payer's patches [55,69–70]. As drug-loaded NLCs pass consecutively through the digestive system, they undergo lipid digestion process step by step. Mainly triglycerides in the lipid nanoparticles are broken down into monoglycerides and free fatty acids (long chain and short chain) due to action of pancreatic enzymes in the duodenum. Released bioactives can either undergo active or passive transport through enterocytes or they may enter lacteals through chylomicron-mediated pathway [71]. In the later pathway, the long-chain fatty acids, monoglycerides and bioactive molecules get surrounded by amphipathic bile salt molecules in intestine chyme and forms ‘micelles’ that are capable of reaching the brush border of absorptive cells of the intestine (enterocytes) via crossing aqueous mucin layer. Monoglycerides, fatty acids and bioactives diffuse out from micelles in the enterocytes leaving micelles behind and further reforming triglycerides inside intestinal cells. These lipid constituents in aggregation with cholesterol and phospholipids form chylomicrons encapsulating bioactives inside them, which finally undergo exocytosis. Large size of chylomicrons (approximately 80 nm) prevents their entry through blood capillaries and thus they enter via lacteals circumventing first pass metabolism of the bioactives associated with them (Figure 4). Particle size of NLCs also plays vital role in intestinal transport, in other words, size below 300 nm is desirable for their transport across the intestine and should be suitably controlled [13].

During GIT passage, NLCs circumventing digestion process can be either conveyed to the portal blood via paracellular route bypassing metabolism due to enterocyte enzymes or can be captured by M cells of Payer's patches delivering NLCs to the lymphatic system [71]. Extensive research carried out by Hu *et al*. on conventional lipid nanoparticles (without any surface modification) to determine the *in vivo* fate and exact absorption mechanism employing water quenching fluorescent probes demonstrated no evidence of absorption of intact nanoparticles through the intestinal membrane after oral administration [72]. However, it has been documented that modification of lipid nanoparticles for instance biotin attachment over their surface or charge modification promotes cellular uptake of nanoparticles via intestinal cells, overcoming the mucosal barrier that otherwise hinders their uptake [31,35]. Also, studies on cell-penetrating peptides (CPPs) coated over the surface of lipid nanoparticles are also based on the hypothesis that they will promote cross of intact nanoparticles across GIT, which gain access in systemic circulation where reticuloendothelial system (RES) system plays role [73]. However, *in vivo* studies on engineered NLCs are not satisfactory and there is necessity of detailed future studies to determine effects of composition, physiochemical properties and surface modification on the fate of lipid nanoparticles.

## Oral bioavailability enhancement by NLCs

Owing to the poor bioavailability of most of the developed drugs, various research groups have worked on designing suitable oral delivery systems for such drugs. NLCs have gained high recognition as suggested by various research reports published so far for drugs, such as isoliquiritigenin, fenofibrate, baicalin, etc. [23,43,47]. Various mechanisms are proposed in this regard and improved bioavailability can be attributed to the suppressed degradation of drugs in GIT; direct uptake of NLCs in GIT; permeation enhancement effect of surfactants employed in formulating of NLCs or avoidance of first pass metabolism [74]. Improved solubilization of drug due to the transformation of its physical state from crystalline to amorphous is one of the other reasons for increased bioavailability [18,75]. Also, entrapment of drug in lipid particles provides shielding against the environment and is beneficial for moisture-sensitive and photodegradable drugs. It has also been documented that nanoparticles ranging from 120 to 200 nm prevent uptake via RES, thus enhancing oral bioavailability of drugs [13,76–77].

### Poorly soluble drugs

Appropriate solubility of drug at the absorption site is necessary for achieving acceptable oral bioavailability. However, more than 40% of new chemical entities coming out from combinatorial screening programs are poorly aqueous soluble, which is a subject of major concern to formulation scientists [3]. NLCs have an inherent GI solubilization capacity due to change of the crystalline form of drug into amorphous form, high dissolution velocity due to their small size and utilization of selective lymphatic transport system [78]. Thus, NLCs have been widely implicated in this arena to facilitate solubilization of various lipophillic agents. Kumbhar and Pokharkar improved the dissolution rate of bicalutamide, an anticancer agent that is poorly water soluble drug (5 mg/l) resulting in low oral bioavailability. The drug release of bicalutamide-loaded NLCs was higher, in other words, 62.08% in 24 h in comparison to a drug suspension, which released only 21.99% of drug [27]. Similarly, better dissolution profile was observed for fenofibrate-loaded NLCs compared with its suspension and presented four-fold improvement in the area under curve (AUC) values in orally administered rats [47]. NLCs of baicalin, a poorly water soluble bioactive also showed 1.9-fold enhanced oral bioavailability in comparison to a drug suspension due to its improved solubilization capacity [23]. Various vegetable bioactives and carrot extract were also encapsulated in NLCs that manifested promising antioxidant and anti-inflammatory activity. The resultant NLCs were homogenous and spherical in shape with a dense appearance (Figure 5). Results of cellular integrity also recommended these nanocariers as a promising trend for the management of disorders in which oxidative stress plays an important role [37].

Lercanidipine HCl is an antihypertensive agent that suffers from low solubility and presents poor oral bioavailability (10%). Lercanidipine HCl-loaded NLCs led to the enlargement in surface area, which resulted in an improved release rate to a receptor compartment (pH 7.4) across the rat stomach membrane in comparison to a drug suspension suggesting a higher dissolution rate of the drug. Moreover, drug-loaded NLCs showed superior antihypertensive activity for 24 h in comparison to drug suspension [55]. Repaglinide belonging to meglitinide class is also a poorly water soluble drug and hence its use is restricted in treatment of diabetes. Administration of NLCs of repaglinide orally to diabetic rats resulted in reduction of blood glucose levels in comparison to its marketed tablet due to its better absorption [54]. Similarly, Abdelwahab *et al*. developed NLCs of thymoquinone for oral delivery using high pressure homogenization technique as an aid to improve its gastroprotective activity. Rats pretreated with thymoquinone-loaded NLCs developed significantly reduced areas of ulcers in stomach in comparison to rats pretreated with plain NLCs against ethanol-induced ulcers via higher modulation of pH of gastric contents [38].

NLCs have also shown improvement in the pharmacokinetic profile of flavanoid drug; isoliquiritigenin having multiple health benefits, but presenting diminished therapeutic efficacy of its oral administration in suspension form due to its lipophillic nature. In this study, NLCs composed of glyceryl monostearate (solid lipid); Miglyol 812 (liquid lipid) and Tween 80 (surfactant) were developed. Higher diffusion of NLCs across the unstirred water layer of intestine epithelial cells facilitated by the presence of hydrophilic surfactant led to augmented values of AUC in comparison to drug solution. Moreover, the small size of NLCs facilitated bioadhesion to intestine membrane, which lead to higher absorption of NLCs [43]. Also etoposide, a cytotoxic agent whose absorption is poor due to its hydrophilic nature and is also a substrate for P-gp was encapsulated in NLCs to surmount its delivery issues [26]. IC_50_ values for drug-loaded NLCs were decreased up to five times in comparison to free drug due to the small size of NLCs that were highly absorbed by cells and released high concentration of drug within cells suggesting the applicability of this delivery vehicle for poorly soluble drugs.

### Stabilization of drugs

An orally ingested substance is exposed to varying conditions in different sections of GIT, which can adversely affect the stability of ingested bioactives. For instance, being a hydrophilic and acid labile compound, ifosfamide degrades at stomach pH and has a limited access to cancerous cells. Also, sintering of this compound on storage decreases its dissolution rate, which also presents problems in its delivery. More recently, ifosfamide was encapsulated inside NLCs having chitosan coating over their surface to provide sustained release of the drug. Formulated NLCs showed only 13% release at pH 1.2 for 6 h, suggesting suitability of NLCs in delivering ifosfamide effectively inside the body [62]. Previously, tamoxifen-loaded NLCs also showed the robustness at different pH of GIT, which was otherwise unstable in GIT [46].

The bioactives are susceptible to different environmental conditions and it is mandatory to protect them from such harsh conditions during storage to provide them better shelf life. For example, lipophillic nutraceuticals, such as luetin, β-carotene or quercetin are obligatory due to their multiple health benefits, but their low aqueous solubility and poor chemical stability during storage presents problems in their oral absorption. To trounce these difficulties, researchers have explored the capability of NLCs for accommodating great amounts of such poorly water soluble bioactives and prevent their premature decomposition [79]. β-carotene has been successfully incorporated into NLCs by solvent diffusion method and analysed for particle size and drug stability. The optimized formulation with desired size range, in other words, 8–15 nm was obtained [41]. More importantly, β-carotene-loaded NLCs were able to prevent the drug degradation (only 0–3% degradation rate) in comparison to unprotected β-carotene. The amount of liquid lipid and the temperature conditions during preparation were identified to be important parameters having an impact on drug stability. The addition of liquid lipid led to slower degradation of the loaded bioactive due to formation of sufficient size particles capable of providing improved retention of β-carotene. Also, optimum temperature conditions were selected as higher temperature led to the production of small NLCs that provided higher diffusion to β-carotene leading to instability. The reactive radicals formed from peroxides in Tween 20 at higher temperature could have contributed to instability of β-carotene. Similarly, use of another carotenoid, in other words, lutein is also hampered in nutraceuticals due to its instability and poor water solubility. Presence of polyene chains in its structure makes it prone to oxidative degradation in the presence of light. In a study, NLCs of lutein were designed using omega-3 fatty acids as a liquid lipid for its suitable encapsulation to fortify foods with advantages of lutein and the liquid lipid itself provided supplementary health benefits. *In vitro* antioxidant study also suggested higher potential of lutein-loaded NLCs [68]. Thus, prevention of physical and chemical degradation of drugs via their encapsulation in NLCs can provide fruitful results. All these studies suggest that NLCs can be employed as an effective carrier for improved oral delivery of nutraceuticals and thus opening new avenues for food industry, however it is necessary to check any undesirable interaction of these NLCs components with other food matrices before incorporating them in foods.

### Overcoming extensive first pass metabolism

Appropriately designed NLCs may act as a delivery vehicle capable of protecting drugs from premature degradation during their transport across GIT due to first pass metabolism. NLCs interact with bile salt in GIT to form mixed micelles, which undergo selective lymphatic uptake, thus bypassing the liver [18]. Also, these mixed micelles can promote luminal solubilization of lipid digestion products and provide concentration gradient for absorption. This versatile capability of NLCs of bypassing liver helps in improving therapeutic efficacy of drugs undergoing extensive hepatic metabolism and decreases their dosing frequency along with its dose related side effects. Various pharmacokinetic studies on anticancer drugs [39,46], anti-inflammatory drugs [25] and antihypertensive drugs [55] have shown that NLCs could provide a prolonged release profile that can extend up to several hours following single oral administration. For example, a flavanoid biochanin A having anti-inflammatory activity and being used as a dietary supplement; when administered orally has a bioavailability of only 1–2% due to its rapid clearance from body by extensive first pass metabolism. Recently, Wang *et al*. reported an increase in the plasma half life of biochanin A from 6 to 21 h after single oral administration in rats when encapsulated in NLCs [25]. In another pharmacokinetic study, 2.71-fold increase in the peak plasma concentration was observed in mice after 72 h following oral administration of tamoxifen-loaded NLCs when compared with the suspension of the drug, which was rapidly cleared within 24 h (Figure 6A). Thus, encapsulation of tamoxifen in NLCs was able to result in sustained release of the drug that could not be achieved in suspension form [46]. Flow cytometric analysis also depicted high uptake of radiolabelled NLCs in MCF-7 cell lines (Figure 6B). The anticancer efficacy study carried out in tumor-bearing mice showed 0% survival of untreated control group receiving placebo, whereas 100% survival was observed in groups receiving tamoxifen-loaded NLCs in doses of 1.5 and 3 mg/kg by oral route. Group receiving drug suspension showed only a 12.5% survival until 40 days. Another drug candidate that is rapidly cleared due to its extensive first pass metabolism, in other words, vinpocetine was also successfully loaded in NLCs composed of Compritol 888 ATO and Miglyol 812N employing high pressure homogenization technique. The pharmacokinetic studies of vinpocetine-loaded NLCs depicted augmented C_max_ (1.918-fold) and AUC values (3.2-fold) in comparison to a drug suspension after single administration in Wistar rats [39]. In a similar manner, the encapsulation of carvedilol in NLCs having 110-nm size and 85.11% entrapment efficiency showed sustained release up to 30 h suggesting their applicability in overcoming its extensive metabolism [28].

Simvastatin is a drug of choice for treatment of hypercholesterolemia and is available as an immediate release formulation. It is poorly water soluble and highly metabolized in intestinal gut and liver and exhibits very short t_1/2_ (2 h), which restricts its clinical use due to need of frequent dosing. After encapsulation of simvastatin in NLCs, AUC values were improved up to 4.87-fold and t_1/2_ was almost doubled in comparison to a drug suspension [18]. Further, radiolabelled NLCs showed higher residence in intestine up to 4 h providing opportunity for a higher absorption of the drug from NLCs. In contrast, drug suspension was seen only for 2 h, suggesting better potential of NLCs in enhancing bioavailability. Similarly, better pharmacokinetic parameters were also obtained with lovastatin-loaded NLCs in comparison to drug solution in orally administered mice [57]. In an attempt to improve solubility characteristics and avoid first pass metabolism. Lin *et al*. combined two novel strategies in a single system, in other words, vinpocetine was incorporated in cyclodextrin complexes that were further incorporated in NLCs matrix and showed 39% higher *in vitro* release at pH 6.8 and 7.4 and superior bioavailability in male rabbits (92%) in comparison to conventional NLCs [50].

### P-gp substrate drugs

P-gp, is a 170 kDa protein belonging to the ATP-binding cassette family and is a well-studied protein, which mainly transports large, hydrophobic, cationic or electrically neutral molecules that can have remarkably dissimilar structures [80]. P-gp is mainly present on the apical side of the epithelial cells in the liver, kidney, pancreas and intestine and in endothelial cells in the brain [81,82]. The significant role of intestinal P-gp in determining oral bioavailability has been demonstrated in rodent studies [83]. It acts as an ATP dependent pump that expels drug molecules back into the GI lumen. Various drugs interacting with P-gp includes actinomycin D, colchicines, daunorubicin, doxorubicin, etoposide, mitomycin C, mithramycin, podophyllotoxin, puromycin, taxol, topotecan, triamterene, vinblastine, vincristine etc. The ability of P-gp to transport these drugs results in lower drug efficacy [7]. Low-oral bioavailability caused due to P-gp may be overcome by rationally designing NLCs that allow the drug to bypass P-gp efflux pump. In an interesting study, it was demonstrated that by modulating characteristics of NLCs, the drugs that are substrates to P-gp could be effectively delivered by oral route. Beloqui and their coworkers studied the transport pathway of different NLCs formulations across caco-2 cells as formulation employing both caveolae- and clathrin-mediated pathway can overcome P-gp efflux instead of formulation employing only caveolae pathway for transport. This may be attributed to the fact that P-gp is localized in caveolae and leads to lower permeability of the drug from formulations utilizing this pathway only due to their higher efflux. *Ex vivo* studies showed that formulation having 247-nm particle size (NLCs B) avoided P-gp efflux as it entered by both pathways, whereas other formulations entered only by caveolae pathway (NLCs A and NLCs C) as depicted in Figure 7A [34]. Moreover, the presence of verapamil, a P-gp inhibitor also did not affect the permeability of NLCs B across cells, whereas permeability of the drug suspension, NLCs A and NLCs C was significantly improved, suggesting inherent capability of formulation B in circumventing P-gp efflux (Figure 7B). In another study, spironolactone, a P-gp substrate drug was effectively delivered orally by the judicial selection of excipients for the formulation of NLCs, in other words, two surfactants were utilized to exploit beneficial properties of both. Poloxamer 188 provided steric stabilization to NLCs in blood and Tween 80 helped in inhibiting P-gp, which favored effective delivery of the drug across intestinal cells. Absolute bioavailability determined via detecting plasma level of metabolite of spironolactone was significantly improved (0.7) in case of drug-loaded NLCs in comparison to drug syrup (0.4) taken as reference in orally administered rabbits. Biodistribution studies also revealed higher accumulation of NLCs in the intestine, which promoted higher absorption of the drug from NLCs [33].

### Surmounting toxicity or adverse effects of drugs

When a drug is administered orally, it enters the systemic circulation and gets distributed in different tissues of the body because of its physiochemical properties, which leads to decreased activity of drug than expected and in turn shows drug-associated toxicity. Therefore, it is a prerequisite for a delivery vehicle to deliver the drug at the site of action and at the same time be safe and should also minimize the adverse effects of drug for the effective treatment of any disease. NLCs have helped in improving treatment of various diseases via encapsulating drugs inside them and actively targeting disease area thus minimizing its potential toxicity. For example, tamoxifen is used to treat cancer and its use is accompanied by systemic and hepatic toxicity. Tamoxifen was integrated into NLCs composed of glyceryl monostearate of stearic acid and labrafil WL 2609 BS. On oral administration of 1.5-mg/kg dose of tamoxifen-loaded NLCs, the mice displayed slower clearance and did not show any increased levels of hepatotoxicity markers, whereas their levels were highly elevated with the same dose of tamoxifen suspension and its marketed formulation suggesting the potential of NLCs in circumventing its toxicity [46].

Oral delivery of tripterine is also associated with adverse affects on GIT and kidneys. Chen *et al*. carried out *in vitro* cytotoxicity studies on Caco-2 cells for validating potential of NLCs in reducing toxicity of tripterine. Encapsulation of tripterine decreased cell killing with an IC_50_ value of 2.11 μg/ml, whereas cells treated with an equivalent concentration of drug solution exhibited higher cytotoxicity. Significant reduction in intestinal toxicity of the drug was also observed, this could be attributed to the controlled release of tripterine after its encapsulation in NLCs [84]. Triptolide has a broad spectrum of activity and is utilized in the treatment of cancer, inflammatory and fertility diseases. However, its rapid absorption and short plasma half-life lead to toxic issues in hepatic, renal and other vital systems of body due to rapid fluctuations in plasma levels. Triptolide was encapsulated in NLCs composed of Compritol 888 ATO and Capryol 90, the nanoformulation was capable of decreasing toxicity of the drug in the liver and kidney up to an oral dose of 650 μg/kg, whereas signs of toxicity were observed in free triptolide administered rats even at lower doses of the drug. Pharmacokinetic studies also revealed higher bioavailability of drug from NLCs in comparison to drug solution [85].

Metabolites of few drugs also lead to adverse affects in the body, which necessitates the development of sustained release system for them. For instance, metabolites of montelukast are known to cause hepatotoxicity during its conventional oral therapy. It is documented that long-chain lipids assure lymphatic uptake of NLCs and high drug concentration in mesenteric lymph nodes from NLCs composed with long-chain lipid, in other words, glycerylmonostearate of stearin has been observed [46]. Therefore, montelukast was incorporated in NLCs, formulated using long-chain triglycerides, in other words, Precirol ATO 5 and Capryol 90, which provided higher lymphatic uptake and avoided the associated side effects by bypassing the hepatic system [53]. Pharmacokinetic studies in rats also indicated improvement in C_max_, mean residence time and AUC values in comparison to drug solution and marketed oral granules demonstrating the high therapeutic potential of NLCs in addition to overcoming its side effects.

### Oral cavity

NLCs have also been employed for effective delivery of drugs in the oral cavity, especially with increasing their residence time in oral mucosa. The mucoadhesive properties of positively charged NLCs have promoted their interaction with negatively charged oral mucosa, thus providing optimal residence in the oral cavity. These systems have been studied to be employed for treatment of various bacterial and fungal infections of the oral cavity. However, considering the lower viscosity of NLCs dispersion, these systems are needed to be incorporated in semisolid preparations, such as hydrogels, to permit their better application in oral mucosa. Recently, miconazole-loaded NLCs have been studied to provide local delivery and controlled release to oral mucosa, which otherwise presents difficulty due to its poor solubility. In this study, the antifungal activity of drug-loaded NLCs was found to be more pronounced in comparison to its marketed formulation having 17-fold higher dose in comparison to NLCs [49]. Similar reports have also been published for clotrimazole- [86] and fluconazole-loaded [87] NLCs, thus rendering NLCs an opportunity to deliver hydrophobic moieties within oral mucosa.

## Engineered NLCs

### Mucoadhesive properties

The tight epithelial cells of the GIT are covered with a hydrophilic and negatively charged protective layer of mucus that restricts the passage of foreign particles across GIT. However, researchers have exploited mucus as a beneficial tool to increase plasma concentration and therapeutic efficacy of drugs via formulating engineered nanoparticles having the capability to anchorage with mucus. Binding of nanoparticles to mucus increases their residence time in GIT, which allows passive transport of drugs leading to their increased absorption. Two different approaches employing physiochemical properties of mucus have been documented to provide mucoadhesion property to nanoparticles (Figure 8A & B). First strategy deals with electrostatic attraction between negatively charged mucus and nanoparticles coated with positively charged polymers, such as chitosan and benzalkonium chloride [30]. Other strategy involves formation of covalent bonds between mucus and thiomers coated over the surface of nanoparticles [88]. These have found wider application in drug delivery as they can resist GIT peristalsis movements due to stronger covalent binding of thiol groups of polymers with cysteine-rich components of mucus [89]. Thiomers also possess the capability to bind to the transmembrane domain of P-gp and thus enabling higher delivery of P-gp substrate drugs [90]. *In vitro* studies revealed that thiomer (cysteine) conjugation onto the surface of docetaxel-loaded NLCs using postinsertion technique led to higher mucoadhesion (81.6%) with mucin in comparison to unconjugated NLCs (51.9%) [76]. Also, significantly higher permeability constant and absorption constant of docetaxel were obtained in the case of thiomer-conjugated NLCs in comparison to drug solution and conventional NLCs during *in situ* intestinal perfusion investigation. Better pharmacokinetic parameters of modified NLCs in Sprague Dawley rats were also in concordance with results of intestinal perfusion study suggesting beneficial effects of engineered NLCs.

### Mucus-penetrating properties

It has been documented that customary rejuvenation of mucus layer by a turnover process hampers bioadhesion process [91]. Therefore, a different strategy via coating/binding of neutral charge polymers to NLCs surface has been implicated in pharmaceutical field to produce mucus-penetrating nanoparticles (Figure 8C). Neutral charge prevents electrostatic attraction between NLCs and mucin and overcomes barrier properties of mucus and helps in transport of NLCs across the mucus membrane to reach the systemic circulation [92]. Literature reveals various research studies in which behavior of NLCs in the body has been modified by preventing their mucoadhesion, which resulted in enhanced oral bioavailability of entrapped drugs and decreasing their dosing frequency. In a recent study, dextran-protamine-coated NLCs with neutral charge were examined for penetration enhancement of NLCs across the mucosal barrier of GIT. *In vitro* drug permeability study across mucus secreting cells (Caco-2/HT 29 MTX) depicted that coating of dextran-protamine to the surface of NLCs could remarkably (twofold) enhance the drug delivery across the cell in comparison to uncoated NLCs [35].

Hydrophilic moieties, such as poly(ethylene glycol) (PEG), also have the capability to penetrate the aqueous mucus layer of the GIT (Figure 8D). PEG coating over the surface of nanoparticles provides hydrophilicity and also circumvents RES uptake of nanoparticles via hindering adsorption of opsonins on their surface, which is one of the major obstacles in delivering drugs to sites other than the liver and spleen [93,94]. Moreover, PEGylation also decreases transendothelial electrical resistance (TEER) values of cells, leading to the improvement of the transport of nanoparticles via paracellular route [95]. Recently, Fang *et al*. observed prolonged-plasma concentration (24 h) in case of docetaxel-loaded PEGylated NLCs in comparison to drug solution, which was very low within 12 h postoral administration to rats as PEGylation prevented opsonin binding to intact NLCs in systemic circulation thus avoiding their macrophage uptake [76]. Wang *et al*. also incorporated an anticancer drug biochanin A in PEG-NLCs with the aim of achieving improved oral bioavailability [66]. Results of a pharmacokinetic study performed in rats and *in vitro* cytotoxicity study carried out over MCF-7 cell lines highlighted the superior performance of biochanin A loaded PEG-NLCs in comparison to drug suspension. Further, oral bioavailability of etoposide-loaded DSPE-PEG-NLCs in rats was also significantly improved when compared with conventional NLCs and drug suspension, which was attributed to their higher uptake due to opening of tight junctions of intestinal membrane by DSPE-PEG and reduced RES uptake [67]. Thus, PEGylation approach augmented the improvement in oral bioavailability of drugs.

### Targeting & biodistribution

ζ-potential is one of the fundamental parameters for assessing colloidal stability, in other words, values of ζ-potential above ±30 mV are the foremost requirement for the electrostatic stabilization due to repulsion between particles [45]. In addition to this, it has been observed that surface charge of nanoparticles has a tremendous effect on tissue permeability and cellular uptake as higher positive or negative ζ-potential allows superior phagocytosis [96–98]. The nanoparticles with cationic surface have been studied widely in oral delivery for increasing the residence time of nanoparticles in GIT as mentioned above [99]. The positive charge of nanoparticles provides their high interaction with the intestinal surface (negatively charged) leading to enhanced absorption of nanoparticles across intestine [100]. Moreover, surface charge of nanoparticles also influences their cellular uptake and biodistribution *in vivo*. Thus, engineered NLCs have been used as a delivery vehicle for targeting drugs to specific tissues along with improving its pharmacokinetic profile (Figure 8E). For example, the effect of positive charge of NLCs intended for oral delivery was investigated on its biodistribution by Liu *et al*. [101]. Cationic NLCs accumulated predominantly in lung, liver and kidney, suggesting their application for diseases related to these tissues. In another study, cationic NLCs were formulated for higher cellular uptake using positively charged surfactant, in other words, CAE (DL-pyrrolidone carboxylic acid salt of l-cocyl arginine ethyl ester) encapsulating montelukast [53]. However, toxicological evaluation of these cationic NLCs is necessary for their clinical uses.

### Active transport

Various nanosystems have been developed to increase their GIT absorption via decorating them with ligands that are either specific to receptors, transporters or specialized cells of the intestine [102]. Ligands employing receptors (vitamins, transferrin, hormone, etc.) or transporters enter through enterocytes, whereas M cells and goblet cells are involved in case of ligands employing specialized intestinal cells. Innovative strategies employing peptidic ligands for increasing absorption of NLCs via an active transport mechanism (Figure 8F) are currently gaining a great deal of attention. Interesting studies using CPPs that can recognize specific receptors on GIT cells have allowed scientists to utilize them as an effective tool for oral delivery of various drugs. Although the exact mechanism of CPPs attached/coated lipid nanoparticles is unknown, but it has been considered that CPPs either encounters receptor mediated pathway, thus facilitating the internalization processes via endocytosis and translocation of attached nanoparticles or enter by disrupting the intestinal membrane [73]. In a study, *in situ* intestinal perfusion model performed in rats showed that peptide-coated NLCs markedly increased absorption of tripterine in the duodenum and jejunum in comparison to noncoated NLCs and tripterine suspension, in other words, 1.5- and 2.9-fold, respectively. The relative oral bioavailability in beagles was also found to be higher for peptide coated NLCs [73]. More recently, biotin-conjugated NLCs showed augmentation in oral bioavailability of oridonin in rats, in comparison to nonmodified ones, which was attributed to receptor mediated transport. Also, permeability coefficient of biotin modified NLCs was significantly higher (threefold) across jejunum due to numerous receptors for biotin in jejunum [31]. Thus, these novel strategies can help in effectively transporting NLCs across the intestinal membrane.

### Toxicity issues

Safety of nanomaterials employed for therapeutic or diagnostic purposes is one of the prime concerns in the modern arena. Numerous *in vivo* reports have been published till now depicting applicability of lipid nanoparticles in oral delivery; however, very few reports demonstrate the safety profile of these systems [103–105]. Reported literature reveals that biodegradable and physiological lipids used in the formulation of NLCs are well tolerated *in vitro* and *in vivo* and minimizes toxicity issues related to polymeric nanoparticles. During *in vitro* cytotoxicity studies, NLCs system depicted no signs of toxicity on Caco-2 cells and cell viability was >90% suggesting their biocompatible nature [84]. Similar results were obtained in another study, where NLCs didn't show any toxicity in BALB/c 3T3 cells [106] or any acute hepatotoxic effects in rats [38]. An oral acute toxicity study of 14 days on zerumbone-loaded NLCs in BALB/c mice of both sexes indicated no behavioral changes and their feed consumption also remained normal. Histological evaluation also showed no signs of toxicity on various organs (lungs, liver, kidney, etc.) at doses of 100 and 200 mg/kg (Figure 9) and LD_50_ dose of NLCs was found to be higher than employed doses, demonstrating safety of this delivery system in oral delivery [40]. In a more defined study, it was observed that the toxicity of NLCs was dependent on the number of NLCs (particles/ml), in other words, various concentrations of NLCs (particles/ml) were incubated with lymphocytes and it was observed that 2.1 × 10^11^ particles/ml lead to decreased viability of cells (55%). NLCs also showed concentration-dependent cytotoxic effects and hemolytic activity [104]. In an interesting study, various lipids and surfactants to be employed for the formulation of NLCs were preliminarily screened via cytotoxicity assay on most appropriate cell lines for validation of basal toxicity (BALB/c 3T3) with the aim to select the formulation ingredients with minimal cytotoxicity [106]. Thus, it becomes imperative to carry out toxicity studies of materials employed for the formulation of NLCs.

## Conclusion & future perspective

The poorly water soluble nature of drugs, resulting from drug discovery processes remains a major concern for pharmaceutical scientists despite their constant hard work. Besides their high *ex vivo* potency, these candidates suffer from poor oral bioavailability and other issues, such as poor permeability, high first pass metabolism, toxicity concerns and instability in GIT. Increase in pubmed data since last decade indicated huge interest of researchers toward lipid-based nanoparticles. Successful NLCs based dermal products (Cutanova Nanorepair Q10 and FloraGlo^®^) introduced on the market have also directed formulation scientists to evaluate their potential in other routes. Researchers have explored the capability of these lipid nanoparticles for accommodating great amounts of bioactives, preventing their premature decomposition and enhancing their oral bioavailability. The various surfactants employed in NLCs formulations can overcome P-gp efflux and could also enhance permeability of drugs across the intestinal membrane by causing disruption in the membrane. Better pharmacokinetic parameters of drug-loaded NLCs obtained during different studies suggested that NLCs can serve as promising tool for enhancing therapeutic efficacy of drugs and also providing controlled release of encapsulated drugs. Different engineered NLCs investigated for further boosting their potential suggested that tuning of physical characteristics of NLCs via changing their composition/method of fabrication could provide fruitful results. Although the exact mechanism underlying behind enhanced absorption by NLCs needs to be addressed and solubility of drugs in different lipids needs to be determined as drugs show limited solubility in various lipids limiting their dose. Thus, further detailed *in vivo* studies are mandatory for taking them to clinics and considering the increased research on this system, it can be concluded that the NLCs based system intended for oral delivery will definitely reach clinical studies in near future.

**Table 1.** **Comparison between properties of solid lipid nanoparticles and nanostructured lipid carriers.**

**Properties**	**SLNs (developed since early 1990s)**	**NLCs (developed since early 2000s)**
Drug encapsulation efficiency	Lesser drug encapsulation efficiency due to formation of highly ordered crystalline arrangement	Higher drug encapsulation efficiency as blend of solid lipids and liquid lipids form disordered structure providing higher space for drug loading Also due to high solubility of drug in liquid lipids
Shelf-life storage	Drug expulsion takes place during storage due to polymorphic transition of high-energy modification formed during fabrication to low-energy modification during storage	No polymorphic transition takes place and drug expulsion is prevented
Release rate	Slower release as mobility of drug in crystalline form is less	Faster release as mobility of drug in crystalline form is high

NLC: Nanostructured lipid carrier; SLN: Solid lipid nanoparticle.

**Table 2.** **Nanostructured lipid carriers fabricated using emulsification and low-temperature solidification technique.**

**Active ingredient**	**Solid lipid**	**Liquid lipid**	**Surfactants**	**Size (nm)**	**Research highlights**	**Ref.**
Baicalin	Glycerol monostearate	Medium chain triglyceride	Soya lecithin	244.7	1.9-fold improvement in AUC and 1.7-fold improvement in mean residence time in case of drug-loaded NLCs in comparison to drug suspension	[23]
Biochanin A	Glycerol monostearate	Medium chain triglyceride	Soya lecithin, Tween 80	148.5 ± 2.88	Higher AUC value of drug-loaded PEGylated NLCs in comparison to drug suspension Biochanin-loaded PEGylated NLCs showed higher *in vitro* cytotoxicity against MCF-7 cell line compared with drug suspension	[25]
Etoposide	Glyceryl monostearate	Soyabean oil	Soya lecithin	91.2–125.9	Higher cytotoxicity of drug-loaded NLCs against human epithelial-like lung carcinoma cell	[26]
Silymarin	Glycerol monostearate	Oleic acid	Tween 80	223.73 ± 43.39	*In vivo* study revealed high accumulation of drug in liver after encapsulation in NLCs	[15]

AUC: Area under curve; NLC: Nanostructured lipid carrier.

**Table 3.** **Nanostructured lipid carriers fabricated using high pressure homogenization technique.**

**Active ingredient**	**Solid lipid**	**Liquid lipid**	**Surfactants**	**Size (nm)**	**Research highlights**	**Ref.**
Bicalutamide	Precirol^®^ ATO5	Triacetin	Phosal^®^ 53 MCT, Pluronic^®^ F-127, sodium taurocholate	130 ± 4.8 to 240 ± 4.1	Precirol^®^ ATO 5 and Triacetin were found to be suitable lipids High entrapment efficiency was achieved	[27]
Carvedilol	Glyceryl mono stearate	Oleic acid	Poloxamer 188, Tween 80	110	Desired particle size and percent entrapment efficiency were obtained for enhancement of oral bioavailability	[28]
Lovastatin	Precirol^®^ ATO 5	Squalene	Myversol 18–04 K	278 ± 2.6	Pharmacokinetic parameters were improved after incorporation in NLCs	[19]
Luteolin	Precirol^®^ ATO 5	Labrasol^®^	Cremophor ELP	43.89 ± 4.5	Luteolin's oral bioavailability improved after encapsulation in NLCs	[29]
Mitotane	Stearic acid	Triacyl glycerol	Tween 80, Span 85	250	NLCs can be successfully provided with positive charge for better mucosal adhesion	[30]
Oridonin	Glycerin monostearate	Medium chain triglyceride	Soyabean lecithin S100, poloxamer 188	144.9	Performance of Biotin-modified oridonin-loaded NLCs in bioavailability was significantly higher in comparison to nonmodified NLCs	[31]
Quercetin	Imwitor 900K	Medium chain triglyceride	Tween 80, lecthin, Span 20	˜34 to 47	Maximum bioaccessbility was observed with NLCs and lipid nanoemulsions compared with SLNs and drug solution	[32]
Spironolactone	Precirol^®^ ATO 5	Miglyol 812	Tween 80, poloxamer 188	150 ± 5	Significant improvement in oral bioavailability of drug in comparison to drug syrup High retention of radiolabeled NLCs in intestinal mucosa	[33]
Saquinavir	Precirol^®^ ATO 5	Migylol 812	Tween 80, poloxamer 188	247	Hydrophilic P-gp substrate drugs can be effectively delivered through oral route after encapsulation in NLCs	[34]
Saquinavir mesylate	Precirol^®^ ATO 5	Migylol 812	Tween 80, poloxamer 188	244 ± 1	Nine-fold increase in saquinavir permeability from Dextran-protamine NLCs in comparison to noncoated NLCs	[35]
Testosterone undecanoate	Dynasan188	Oleic acid	Tween 80	200	AUC values were found to be doubled in comparison to commercial formulations	[36]
Thistle oil, safflower oil, sea buckthorn oil, carrot extract	Glycerol monostearate	Cetyl alcohol, beeswax	Tween 20, phosphatidyl choline	70–140	NLCs demonstrated better antioxidant and anti-inflammatory properties due to synergistic effect of plant-derived oils and extract	[37]
Thymoquinone	Softisan^®^ 154	Olive oil	Polysorbate 80	75 ± 2.4	Improved gastroprotective effects of drug Pharmacokinetic properties of drug were improved after encapsulation in NLCs	[38]
Vinpocetine	Compritol 888 ATO	Migylol 812	Lecithin solutol HS-15	136 ± 4.2	Improved oral bioavailability of drug-loaded NLCs (322%) in comparison to drug suspension	[39]
Zerumbone	Hydrogenated palm oil	Olive oil, lipoid S100	Tween 80, thiomerasol	52.68 ± 0.1	NLCs showed no acute toxicity at oral doses of 100 and 200 mg/kg	[40]

AUC: Area under curve; NLC: Nanostructured lipid carrier; SLN: Solid lipid nanoparticle.

**Table 4.** **Nanostructured lipid carriers fabricated using solvent diffusion technique.**

**Active ingredient**	**Solid lipid**	**Liquid lipid**	**Surfactants**	**Size (nm)**	**Research highlights**	**Ref.**
β-carotene	Palmitic acid	Corn oil	Tween 20	8–15	Decreased β-carotene degradation was achieved	[41]
Docetaxel oleate	Glyceryl monostearate	Oleic acid	Poloxamer F68	100	Prodrug of docetaxel, in other words, docetaxel oleate was successfully loaded with high loading efficiency NLCs showed 2.06-fold higher bioavailability in comparison to drug suspension	[42]
Isoliquiritigenin	Glycerol monostearate	Miglyol^®^ 812	Tween 80, poloxamer 188	NA	Significant improvement in AUC values in orally administered drug-loaded NLCs in comparison to oral solution of drug	[43]
Raloxifene hydrochloride	Glyceryl monostearate	Capmul MCM C8	Polyvinyl alcohol	32.50 ± 5.12	3.75-fold significant improvement in bioavailability of poorly soluble drug than plain drug suspension, which bestow its potential role as suitable carrier system for oral delivery in the treatment of osteoporosis	[44]
Tamoxifen citrate	Glyceryl monostearate	Labrafil WL 2609 BL	Tween 20, Tween 80, poloxamer 188	215.60 ± 7.8	Improved pharmacokinetic parameters of drug-loaded NLCs in comparison to drug suspension Improved anticancer efficacy	[45]
Tamoxifen citrate	Glyceryl monostearate	Labrafil WL 2609 BL	Tween 20, Tween 80, poloxamer 188	215.60 ± 7.8	NLCs were able to withstand various GIT media Sustained release was obtained irrespective of pH of media	[46]

AUC: Area under curve; GIT: GI tract; NA: Not available; NLC: Nanostructured lipid carrier.

**Table 5.** **Nanostructured lipid carrier fabricated using ultrasonication technique.**

**Active ingredient**	**Solid lipid**	**Liquid lipid**	**Surfactants**	**Size (nm)**	**Research highlights**	**Ref.**
Domperidone	Trimyristin	Cetyl recinoleate	Soy phosphatidyl choline, Tween 80	30.45	*In vitro* release studies demonstrated controlled release of drug over 24 h NLCs were stable during 40 days storage period of time	[21]
Fenofibrate	Compritol 888 ATO	Labrafil M 1944CS	Tween 80, soya lecithin	89.9 ± 4.9	Four-fold improvement in AUC	[47]
Lutein	Precirol^®^ ATO 5	Corn oil	Pluronic F 68, Myverol 18–04 K	130	Improvement in aqueous solubility of lutein after encapsulation in NLCs Sustained release of lutein was achieved	[48]
Miconazole	Gelucine^®^ 43/01	Miglyol^®^ 812	Benzlalkonium chloride, Tween 80	≈200	Drug loaded in NLCs showed similar therapeutic efficacy to the marketed formulations having 17-fold higher dose of drug	[49]
Vinpocetine	Compritol 888 ATO	Migylol^®^ 812	Solutol HS-15	89 ± 21	Relative bioavailability of solid vinpocetine-cyclodextrin tartaric acid-loaded NLCs was 592% higher in comparison to vinpocetine suspension and 92% in comparison to conventional NLCs	[50]

AUC: Area under curve; NLC: Nanostructured lipid carrier.

**Table 6.** **Nanostructured lipid carriers fabricated using emulsification-sonication technique.**

**Active ingredient**	**Solid lipid**	**Liquid lipid**	**Surfactants**	**Size (nm)**	**Research highlights**	**Ref.**
Fenofibrate	Precirol ATO 5	Captex 100	Tween 80	227.5	Fluid bed coating was found to be suitable method for solidification of NLCs without affecting their *in vivo* performance AUC values were improved after encapsulation in NLCs in comparison to marketed capsule	[51]
Iloperidone	Lauric acid	Phosal^®^ 53 MCT	Tween 80, poloxamer 188	160	8.3-fold improvement in oral bioavailability of iloperidone after encapsulation in NLCs in comparison to drug suspension	[52]
Montelukast	Precirol^®^ ATO 5	Capryol 90	D-L pyrrolidine carboxylic acid salt of l-cocyl arginine ethyl ester	181.4 ± 6.5	Single oral dose of NLCs in rats provided 143-fold improvement in bioavailability in comparison to montelukast-aqueous solution	[53]
Progesterone	Stearic acid	Oleic acid	Tween 20	326.7	Sustained release of progesterone for 24 h was obtained	[20]
Repaglinide	Precirol^®^ ATO 5	Caproyl 90	Gelucire 50/13	170–210	Novel stabilizer (Gelucire 50/13) was investigated for first time Higher antidiabetic action of repaglinide-loaded NLCs in comparison to commercial repaglinide tablets	[54]

AUC: Area under curve; NLC: Nanostructured lipid carrier.

**Executive summary**

**Background**

Nanostructured lipid carriers (NLCs) are promising oral delivery vehicle due to their higher stability during storage and ease of scalability without any requirement of sterile conditions.
These systems are capable in entraping both hydrophilic and lipophilic drugs.
Biocompatible and biodegradable lipids employed in their fabrication minimize toxicity issues.

**NLCs for oral delivery**

Various studies have been reported based on NLCs for overcoming solubility, permeability, stability and toxicity issues of bioactives.
Different engineered NLCs for generating mucus adhesive, mucus-penetrating particles or for better targeting have been explored.
Peptidic ligands are also finding wider applicability in oral delivery as they facilitate active transport of the drugs loaded inside NLCs across the intestinal membrane.
Better pharmacokinetic parameters of drug-loaded NLCs have been obtained during different studies.
Further, significant *in vivo* studies addressing their mechanism underlying enhanced oral bioavailability are needed for bringing these systems in clinics.
